# Implementation of integration strategies between primary care units and a regional general hospital in Brazil to update and connect health care professionals: a quasi-experimental study protocol

**DOI:** 10.1186/s12913-016-1626-9

**Published:** 2016-08-12

**Authors:** Mario Maia Bracco, Ana Carolina Cintra Nunes Mafra, Alexandre Hannud Abdo, Fernando Antonio Basile Colugnati, Marcello Dala Bernardina Dalla, Marcelo Marcos Piva Demarzo, Ises Abrahamsohn, Aline Pacífico Rodrigues, Ana Violeta Ferreira de Almeida Delgado, Glauber Alves dos Prazeres, José Carlos Teixeira, Silvio Possa

**Affiliations:** 1 Hospital Municipal Dr. Moysés Deutsch, M’Boi Mirim, São Paulo, Brazil; 2 Hospital Israelita Albert Einstein, São Paulo, Brazil; 3 School of Medicine, University of São Paulo, São Paulo, Brazil; 4 Garoa Hacker Club, São Paulo, Brazil; 5 School of Medicine, Federal University of Juiz de Fora, Juiz de Fora, Brazil; 6 Secretariat of Health of Espírito Santo State, Espírito Santo, Brazil; 7 Superior School of Sciences of Santa Casa de Misericórdia of Vitória - EMESCAM, Vitória, Brazil; 8 Paulista Medical School, Federal University of São Paulo (UNIFESP), São Paulo, Brazil; 9 Center of Studies and Research Dr. João Amorim – CEJAM, Porto Alegre, Brazil

**Keywords:** Health system integration, Evidence-based implementation, Health professional education, Interdisciplinary communication, Primary health care

## Abstract

**Background:**

Better communication among field health care teams and points of care, together with investments focused on improving teamwork, individual management, and clinical skills, are strategies for achieving better outcomes in patient-oriented care. This research aims to implement and evaluate interventions focused on improving communication and knowledge among health teams based on points of care in a regional public health outreach network, assessing the following hypotheses: 1) A better-working communication process between hospitals and primary health care providers can improve the sharing of information on patients as well as patients’ outcomes. 2) A skill-upgrading education tool offered to health providers at their work sites can improve patients’ care and outcomes.

**Methods/Design:**

A quasi-experimental study protocol with a mixed-methods approach (quantitative and qualitative) was developed to evaluate communication tools for health care professionals based in primary care units and in a general hospital in the southern region of São Paulo City, Brazil. The usefulness and implementation processes of the integration strategies will be evaluated, considering: 1) An Internet-based communication platform that facilitates continuity and integrality of care to patients, and 2) A tailored updating distance-learning course on ambulatory care sensitive conditions for clinical skills improvements.

The observational study will evaluate a non-randomized cohort of adult patients, with historical controls. Hospitalized patients diagnosed with an ambulatory care sensitive condition will be selected and followed for 1 year after hospital discharge. Data will be collected using validated questionnaires and from patients’ medical records. Health care professionals will be evaluated related to their use of education and communication tools and their demographic and psychological profiles. The primary outcome measured will be the patients’ 30-day hospital readmission rates. A sample size of 560 patients was calculated to fit a valid logistic model. In addition, qualitative approaches will be used to identify subjective perceptions of providers about the implementation process and of patients about health system use.

**Discussion:**

This research project will gather relevant information about implementation processes for education and communication tools and their impact on human resources training, rates of readmission, and patient-related outcomes.

**Electronic supplementary material:**

The online version of this article (doi:10.1186/s12913-016-1626-9) contains supplementary material, which is available to authorized users.

## Background

In structured societies, better care for all must be delivered in a timely and effective manner. Universal health systems face shortages of funding, increasing patient demand, and more complex treatment options [1]. The Brazilian Unified Health System (Sistema Único de Saúde, SUS) aims for the provision of universal access to the population, prioritization of equity in the health services, and integrality across assistance levels [2].

The Regional Healthcare Attention Network (RHAN) is the result of a public policy that has been incorporated in the SUS. RHAN is a framework recommended by the Brazilian Ministry of Health to improve the coordination among the levels of the health care system [3]. The core idea of RHAN is that better communication and the use of evidence-based information by health teams at different points of care should result in coordination and integration in terms of how patients are viewed and managed [4]. Primary care has the most relevant role in RHAN’s coordinating efforts to assess patients who should be referred to the other levels of care (e.g., specialists and hospitals), but patients receiving treatment at other hierarchical levels are also received and managed by the primary care sector [5].

Effective communication among teams based in hospitals and those based in primary care units (PCU) is an essential factor for RHAN functioning regarding patient safety and continuity of care. Hospital discharge summaries have been the main tool used to accomplish these tasks. Frequently, these discharge summaries are incomplete, lacking critical information, and of poor quality. Additionally, they sometimes fail to reach the PCU teams in a timely manner. This lack of communication and information exchange can increase the risk of hospital readmission and lead to discontinuity in patient care [6–9]. Hospitalization rates for ambulatory care sensitive conditions (ACSC) have been inversely correlated with the effectiveness of primary care attention. However, there is currently no consensus on which diseases or conditions should make up the ACSC lists, as these can vary between countries and regions. The Brazilian Ministry of Health developed an ACSC list that includes chronic and infectious diseases [10].

Another critical point for RHAN is the implementation of evidence-based recommendations into routine clinical practice, evidence that can lead to better clinical outcomes for patients [11]. Nevertheless, there is some resistance from health professionals to adhering to evidence-based protocols, because many of these protocols would not be applicable to the actual working conditions of these professionals—the so called “real world” assistance setting. A further obstacle is that many protocols contain too much information and need to be synthesized [12, 13]. Keeping health professionals updated on standards of practice based on patients’ needs is also a challenge. A recent systematic review on continuing medical education in the United States concluded that keeping informed of developments in their field, improves physicians’ performance and patients’ health outcomes, with the positive effect on physicians’ performance being more reliable [14]. Despite the positive impact of continuing medical education, the extent of this effect is unknown, underscoring the need for a debate on effective strategies to keep health professionals updated [15]. The desired health professional profile for the 21st century will synthesize the available information and resources to make better patient management decisions. In pursuit of this goal, several new technologies can be applied to offer information and support, and to improve teamwork [16].

To overcome the fragmentation of health care, which creates a burden for both patients and the health system, knowledge and communication management should help to build capacity among health professionals, empowering them to move toward transformative action. This article describes the protocol for the implementation of integration strategies based on innovative approaches to improve communication and knowledge refinement among health professionals, as well as the study protocol for the short-term impact evaluation of this implementation effort.

## Methods/design

### Research setting

Hospital Municipal Dr. Moyses Deutsch (HMMD) is a large municipal government-funded general hospital (300 beds) located in the south of São Paulo City (M’Boi Mirim District). The hospital is managed by Hospital Israelita Albert Einstein (HIAE) under a public–private partnership. It is the unique referral hospital for 600,000 people who are registered in 31 PCUs covered by the primary care teams. For the planned study, the 18 PCUs managed by the Center of Studies and Research Dr. João Amorim (CEJAM), a non-government organization that manages the primary care under a public–private partnership framework, will be selected as a convenience sample. Table 1 provides an overview of the study objectives and their associated methods.

**Table 1:** Overview of study methods by objective

Element	Objective 1	Objective 2	Objective 3	Objective 4
Objective	To implement and evaluate an Internet-based platform that links health providers at a public hospital to18 primary care units (PCU) to improve communication among health services	To promote and evaluate a distance-learning course (DLC) to update health professionals on the clinical management of ambulatory care sensitive conditions (ACSC)	To verify the impact of the two strategies (communication and course) on patients’ hospital outcomes (30-day readmission rateslength of stay and mortality)	To verify the long-term impact of the two strategies (communication and course) through the follow-up of the patient cohort
Design	Longitudinal study of communications among health services	Cross-sectional study of the course evaluation and longitudinal study of enrolled health care providers	Longitudinal study of patients with ACSC using hospital electronic medical records	Longitudinal cohort study
Procedures	1. Platform development 2. Registration and training of professionals 3. Use the platform in the following ways: i. The hospital sends an admission warning and a structured questionnaire on patient’s information to the PCU ii. The PCU manager responds with information about the patient iii. Hospital nurses enter the information in the patient’s electronic medical record iv. The hospital sends the discharge summary to the PCU	The course consists of five modules and uses discussion forums, case studies, web conferences, and face-to-face meetings Information is obtained from the students and the DLC platform	This phase uses patient data collected from hospital electronic medical records	Selection of eligible patients among all ACSC hospitalized patients Administration of questionnaires by the research team 1-year follow-up at the PCU or the patient’s home Gathering of information from the PCU’s paper-based medical records, covering at least 1 year before and 1 year after the hospitalization. Selection of eligible patients for telemedicine consultation Training of all interviewers, inter-rater agreement analysis
Participants/ Research units	Communications among the health providers and the research team	Health care providers who signed the informed consent form agreeing to volunteer for this research	All hospital admissions of adult patients who had at least one ACSC from 2013 to 2017	Adult patients admitted to the hospital with an ACSC who are registered at any PCU enrolled in the study and are able to respond to questionnaires and sign the informed consent form
Measures	Frequencies of alerts sent by the hospital, answers sent by the PCU, patients’ information being included in the electronic medical record, discharge summaries being sent by the hospital to the PCU Duration of time between each of these steps	DLC: Frequencies of students’ access to didactic materials, participation in forums and face-to-face meetings Professionals: sociodemographic characteristics, quality of life, mindfulness, clinical types of burnout, and primary care attention attributes.	Patients: Main and secondary diagnoses, address, length of stay, discharge type, readmissions, episodes in the emergency department, reference PCU Medical record audits: Content quality of diagnosis, anamnesis, physical exam, lab exams, discharge summary, nurse registers	Patients’ information at inclusion: Sociodemographic characteristics, quality of life, primary care attention attributes. Patients’ information at follow-up: Same as in inclusion, plus PCU medical record information, geographical location of residence, consultations, medications taken, main outcomes (therapeutic plan adherence, disease complications, health services access, mortality, readmission in any hospital) Telemedicine: Reason for telemedicine request, use of diagnosis or treatment information to help with decision making, patients’ obtained outcomes PCU medical record audits: Content quality of registered information
Sample size	Not applicable	All health care providers who accept enrollment in the DLC, of eligible health care providers from the hospital and PCU health teams	All eligible patients during the study period. In total, 3422 patients with at least one ACSC were admitted from 2013 to 2014	560 patients, based on the observed incidence of 16 % readmission within 30 days, fitting a valid logistic model with up to nine covariates. Each interviewer will collect data from at least 10 patients and will be paired with another interviewer to assess inter-rater agreement
Data analysis	Absolute and relative frequencies and median and interquartile ranges with 95 % confidence intervals. Chi-square and Mann–Whitney tests to compare measures among implementation phases Social network analysis for assessing the communication structure	DLC: Absolute and relative frequencies, chi-square tests to compare measures from each module Professionals’ information: Summary measures with 95 % confidence intervals, generalized linear models to assess possible associations, generalized estimating equations to compare results over time	Patients: Summary measures with 95 % confidence intervals, generalized estimating equations for length of stay, logistic models for mortality, kernel density to assess geographical location through patients’ addresses Audits: Frequencies of adequacy of registered information with 95 % confidence intervals, chi-square tests for comparisons over time, inter-rater reliability of auditors by percent agreement and Gwet’s agreement coefficient	Baseline information: Summary measures with 95 % confidence intervals, hypothesis tests and/or generalized linear models to assess possible associations in the collected data Main outcomes (readmission rates): Summary measures with 95 % confidence intervals, generalized linear models to assess associations, generalized estimating equations to compare results over time Inter-rater agreement: Percent agreement and Gwet agreement coefficient

### Interventions

The strategy comprises an Internet-based platform developed as a social network tailored to health professionals’ use that allows the sharing of patients’ information, documents, and treatment plans between the hospital and 18 PCUs. In addition, telemedicine support will be made available for patients who, for any reason, do not receive specialist services. An electronic knowledge platform will also be developed to keep the health professionals updated through a distance-learning course (DLC) in clinical management focused on diseases that are part of the Brazilian ACSC list.

- Communication platforms
  An Internet-based social network that was developed using free software (based on the open source project Hubzilla [17], formerly known as Redmatrix) and was tailored for this intervention will be made available to the HMMD and PCU health teams [18]. This platform will host private communications among the health providers about their common patients. This shared information will flow through secure channels using the HTTP Secure (HTTPS) protocol to ensure that patients’ information can only be accessed by the appropriate health professionals, whose identity will be authenticated by their credentials (login and password).
  The platform’s interface includes a hospitalization alert that will ask for specific information that can help the hospital teams to manage the patient during their stay. The responses from the PCU will be incorporated into the hospital electronic medical record. Afterwards, the discharge summary and prescriptions will be delivered to the PCU through a similar discharge alert. Beyond the sharing of information, each alert allows for an online asynchronous conversation to develop between the professionals at each end.
  Additionally, a telemedicine service (already implemented at HMMD) will be made available to those patients followed during the study, who, for any reason, do not reach specialized services in the SUS. For example, long waiting times for consultations or more sophisticated exams, patient’s lack of resources to pay for transportation to the point of care, or simply absenteeism are situations that can hinder treatment plans, potentially harming patients’ clinical outcomes. Eligible patients will be referred by the primary care teams. These patients will then visit HMMD, where they will be seen by an HIAE physician or nurse specialist via telemedicine equipment. This is an ongoing project supported by the HIAE and the Ministry of Health, with benefits reported among patients seen in the HMMD emergency room [19].
- Clinical management updating DLC
  A DLC based on the Brazilian ACSC criteria, focusing on physicians, nurses, and multi-professional staff, will be implemented. The DLC will be hosted by the HIAE Teaching Department’s structured platform, Taleo™. This knowledge platform was conceived to transmit and record web conferences, host discussion forums, and store and transfer archives. Other educational resources, such as structured clinical cases, will be developed to illustrate situations faced by health providers in real-world scenarios.
  The DLC will be divided into five modules, each 8 weeks in duration, covering the Brazilian ACSC list: respiratory, cardiovascular, diabetes, gastroenterology and mixed infectious diseases, and major health risk factors (smoking, alcoholism, drugs, pain, nutrition, and physical inactivity). Web conferences will be transmitted and recorded on a weekly basis. The 18 PCUs and HMMD will participate in the web conferences, which will feature a presentation on an ACSC by a professional expert speaker and a professional generalist’s comments on specific issues to provide participants with updated evidence-based knowledge. A tutor will encourage discussions in the forum, using a structured clinical case as a tool to demonstrate the applicability of the scientific evidence. The use of the ACSC-related guidelines and/or of evidence-based protocols will be emphasized. After each module, a face-to-face meeting will be carried out to emphasize teamwork and clinical decision-making processes, wrapping up the module content with mixed discussion groups tasked with generating proposals from the health providers for applying evidence-based recommendations at their care facilities. The DLC participants will be evaluated based on their participation in the discussion forums, weekly quizzes, and a final exam at the end of each module. Additionally, the participants will use a structured questionnaire to evaluate each module in terms of their satisfaction with the DLC structure, content, relevance to clinical practice, and feasibility of application in the workplace [see Additional file 1].

### Sample and study design for impact evaluation

- Patients
  The impact evaluation of the integration strategy will use a quasi-experimental cohort with historical controls design. Participants will be adult patients hospitalized for ACSC who will be followed up 1 year after their hospital discharge. Patients’ data will be compared with data on the same patients from different time periods as historical controls, using medical records dating at least 1 year before and 1 year after the patients’ inclusion in the study.
  The sample of patients will be selected in two stages. First, a convenience sample of 18 PCUs will be selected, as described above. In the second stage, 560 hospitalized adult patients living in the territories covered by and registered in the selected PCUs will be selected based upon their main or secondary diagnosis, which must fit the Brazilian ACSC criteria, excluding female pelvic disorders and pregnancy-related diseases (Table 2). The calculation of this sample size was based on the 2013–2014 HMMD incidence of 30-day readmissions for ACSC, which was 16 %. This sample size will permit a valid logistic model with up to nine covariates to be fit [20].

**Table 2:** Diagnoses included in this study and their International Classification of Diseases (ICD-10) codes

Diagnosis	ICD-10 code
Infectious gastroenteritis and complications	E86, A00–A09
Gastrointestinal ulcer	K25–K28, K92.0, K92.1, K92.2
Ear, nose, and throat infections	H66, J00–J03, J06, J31
Bacterial pneumonias	J13–J14, J15.3–J15.4, J15.8–J15.9, J18.1
Diseases of the lower airways	J20, J21, J40–J44, J47
Asthma	J45–J46
Tuberculosis	A15.4–A15.9, A16.3–A16.9, A17.1–A17.9
Pulmonary tuberculosis	A15.0–A15.3, A16.0–A16.2
Heart failure	I50, J81
Hypertension	I10–I11
Angina pectoris	I20, I24
Cerebrovascular diseases	I63–I67, I69, G45–G46
Diabetes mellitus	E10–E14
Infection in the kidney and urinary tract	N10–N12, N30, N34, N39.0
Epilepsies	G40–G41
Syphilis	A51–A53
Infection of the skin and subcutaneous tissue	L01, L02, L03, L04
  Exclusion criteria are being under palliative care or dying during the first hospitalization, as well as being unable to respond to the questionnaires because of a cognitive deficit or an unstable clinical condition. The data of all eligible ACSC patients identified by the research team will be analyzed according to their diagnosis, length of stay, outcomes (discharge, death, or transfer to another hospital), and readmission rates.
  The Brazilian validated version of the Primary Care Attention Tool [21], the World Health Organization Quality of Life (WHOQoL Brief) [22], and a sociodemographic questionnaire [see Additional file 2] developed by the research team based on other validated instruments [23–27] to collect data on social and environmental characteristics will all be administered at the bedside after the patient has signed the informed consent form, and again at the follow-up visit in the patient’s home or PCU, as chosen by the patient. At the follow-up, an additional questionnaire about treatment plan adherence [see Additional file 3], disease monitoring, and access to all levels of the health services will also be administered.
  Data on the selected patients will be gathered from their medical records and the electronic system of the São Paulo Municipality (SIGA SAÚDE), which registers all patient visits to the points of care managed by the São Paulo municipal health system, as well as prescribed medications. From both HMMD and the PCUs, medical record data will be collected covering the time period at least 1 year before and 1 year after hospitalization, following the historical control design chosen for this study, to search for evidence related to the number of consultations, health promotion and education actions, and access to the other levels of the SUS. Several telemedicine-related variables will also be collected: the reason for the consultation, diagnostic or therapeutic aid, information use in decision making, and patient’s obtained outcomes. All data collected by the research team will be submitted to an inter-rater reliability analysis using Gwet’s coefficient [28].
  At the follow-up visit, some interviews will be video recorded, for patients who authorize the use of their images, to obtain additional information that will be analyzed qualitatively about their use, personal impressions, and satisfaction with the health system. All interviews will be transcribed and coded through subsequent qualitative analysis, informed by the discourse of the collective subject theory [29]. All of the participating patients’ addresses will be mapped using the free software and open data service Open Street Map [30] assess correlations between social and environmental vulnerabilities and patient’s health status.

### Health professionals

The eligible health providers, physicians, nurses, and multi-professional teams, from HMMD and the PCUs, will be invited to participate as research volunteers. Thus, there is no sample size calculation. The eligible professionals are members of 85 primary care teams from the PCUs (85 physicians, 85 nurses, 12 multi-professional staff members, and 20 PCU managers) and 43 physicians, 73 nurses, 29 multi-professional staff members, and 12 medical residents from the general clinic wards of HMMD, summing to 359 eligible providers in total.

Data on sociodemographic characteristics, educational background, quality of life, burnout, and mindfulness will also be collected. As was described above for patients, or health professionals participating in the study, the Primary Care Attention Tool - Professional Version [21] (only for PCU providers), the World Health Organization Quality of Life (WHOQoL Brief) [22], and the sociodemographic and educational background questionnaires developed by the research team [see Additional file 4] will be administered. The Burnout Clinical Subtypes Questionnaire (BCSQ-12) [31] and the Mindfulness Awareness Attention Scale (MAAS) [32] will also be administered to this group. All questionnaires will be self-administered. Data will also be collected from these professionals on their adherence, participation, and use of acquired knowledge in clinical practice, as well as on their evaluation of the course modules. The sources of these data will be the number of accesses to the knowledge portal for the DLC course and qualitative information from focus groups and the discussion forums.

### Patients’ medical records

To ensure the quality of the data that will be gathered from the medical records, a sub-project will be carried out using the Lean and Six Sigma techniques to improve the quality of the information registered by the health providers. The Lean Six Sigma approach has been used to aggregate value in different processes, reducing waste and adding more uniformity and precision through the application of statistical tools [33, 34]. These principles have been recommended for application in clinical and translational research [35]. This project will be focused on the health records review boards of HMMD and the PCUs, and it will be carried on in partnership with the Process Improvement Department of the HIAE. Requirements for the quality criteria for information in the medical records will be defined and aligned as part of the study, to be audited by the members of these review boards. Additionally, inter-rater reliability analyses will be conducted among the auditors on a regular basis.

### Data analysis plan

All variables will be summarized by absolute and relative frequencies or means and medians. Standard deviations, interquartile ranges, and 95 % confidence intervals will also be reported. Possible associations will be evaluated by hypothesis tests and/or generalized linear models and generalized estimating equations to compare the results over time. Inter-rater agreement will be measured by percent agreement and Gwet’s agreement coefficient [28] for the patients’ questionnaires and audits of the medical records. The communication flow among points of care will be measured and analyzed using standard tools from social network analysis. The spatial distribution of the geographical location of patients’ residence will be mapped, and kernel density will be estimated. The data analysis will be performed using R software [36], and we will adopt a significance level of 5 %.

### Study status

At this time, the Internet-based platform has been fully implemented. The DLC was conducted from July 2014 to July 2015. All data related to the health providers have been collected. The Lean Six Sigma project was completed in March 2016. The patients’ selection and inclusion began in November 2014 and was completed in March 2016. Patient follow-up visits are underway. The telemedicine consultations began in April 2016.

## Discussion

There are multiple potential difficulties in adopting an innovation that involves changes in working processes in the health services. According to implementation science models, certain institutional and personal attributes enhance culture, environment, and teamwork, as well as the reaction of these things to inner and outer pressures [37–39]. In general, these innovations demand behavior change among health teams in management and assistance tasks. Adding a new task in addition to the existing ones is a matter of concern in health services in terms of work overload and prioritization of actions concerning relevance to patient care. The incorporation of evidence-based practices tends to compete with previously established processes already being used by health providers, thus also requiring organizational adaptations [40]. Creativity and the rational use of available technology can be useful in tackling fragmentation of care, improving patient outcomes, and lowering health-related costs, all of which are urgent needs.

In the Brazilian health system, the biggest universal system in the world in terms of population and territorial coverage, an efficient RHAN is a necessity that is being built. Nevertheless, it is a difficult task to accomplish. The social network platform described in this protocol will be incorporated through the development of work processes and flows involving the HMMD and PCU teams. This project will gather information about obstacles and critical situations that affect them. The DLC seeks to introduce an educational tool into health workers’ routine that is necessarily easy to access, useful, and feasible, and through which relevant information can be reached and applied in clinical practice. The DLC will be tested and improved based on input from the health provider volunteer participants, who will evaluate certain aspects of the DLC related to access, content, and educational resources. The health professionals’ profiles related to burnout, quality of life, mindfulness, and sociodemographic and educational characteristics will contribute information about how the organization of the SUS affects their work possibilities and, ultimately, their overall health. This study also aims to shed light on feasible options for testing the effectiveness of communication and capacity building strategies for health providers, as well as tools designed to improve these capabilities, provide recognition, and empower the health providers.

The study design is mainly focused on the implementation processes of the interventions to be tested among health professionals, including assessments of the effects of obstacles, resistances, new skills, and new process developments on health teams’ communication and on the availability of evidence-based scientific knowledge. The originality of this study is found in its assessment of a DLC administered to health professionals providing care to a shared group of patients over different points of care and in the implementation of an Internet-based communication platform as a social network. The hospitalized patient-selection process will allow the study to include members of the population who are socially and environmentally vulnerable. The major strength of this study is its status as a pragmatic intervention conducted in a real-world setting. However, there will be methodological limitations in terms of isolating the effects of the proposed interventions. A comprehensive strategy to implement RHAN must consider not only technological innovation and its results, but all features that can interfere with the necessary steps to achieve consolidated changes in work processes and patients’ outcomes. Data quality, safe information channels, and a healthy workforce are themes that are investigated in this research project.

## Abbreviations

ACSC, ambulatory care sensitive condition; CEJAM, Center of Studies and Research Dr. João Amorim (Centro de Estudos e Pesquisas, Dr. João Amorim); CONEP, National Research Ethical Commission (Comissão Nacional de Ética em Pesquisa) ; DLC, distance-learning course; FAPESP, São Paulo State Research Agency (Fundação de Amparo à Pesquisa do Estado de São Paulo); HIAE, Hospital Israelita Albert Einstein; HMMD, Hospital Municipal Dr. Moyses Deutsch - M’Boi Mirim; PCU, primary care units; PPSUS, Research Program for the SUS (Programas de Pesquisa para o SUS); RHAN, Regional Healthcare Attention Network; SIGA SAÚDE, Municipal Integrated System for Health Assistance Management  (Sistema Integrado de Gestão da Assistência à Saúde) ; SUS, Brazilian Unified Health System (Sistema Único de Saúde)

## Additional files

Additional file 1: Questionnaire of Distance Learning Course – Professional’s Assessment. (DOCX 29 kb) Additional file 2: Sociodemographic patient’s questionnaire. (DOCX 50 kb) Additional file 3: Questionnaire on treatment adherence. (DOCX 72 kb) Additional file 4: Socio-demographic health professional questionnaire. (DOCX 38 kb)
