# Supplementary material for: Implementation of integration strategies between primary care units and a regional general hospital in Brazil to update and connect health care professionals: a quasi-experimental study protocol
Source: BMC Health Serv Res. 2016 Aug 12;16:380. doi: 10.1186/s12913-016-1626-9 (PMC4983016; doi:10.1186/s12913-016-1626-9)
Supplement: Additional file 1: — Questionnaire of Distance Learning Course – Professional’s Assessment. (DOCX 29 kb) [file 12913_2016_1626_MOESM1_ESM.docx]

Questionário de Avaliação do Curso EAD

Questionnaire of Distance Learning Course – Professional’s Assessment

1. O que fez você interessar-se em fazer o curso? (pode marcar mais de uma opção)

- (  ) Prestar melhor assistência à população
- (  ) Integração entre Hospital e Atenção Básica
- (  ) Aprendizado, conhecimento ou atualização pessoal
- (  ) Aplicabilidade na prática clínica
- (  ) Conteúdo do curso
- (  ) Formato do curso
- (  ) Outro. Qual?________________________________________________

What made you most interested in taking this course? (you can choose more than one option)

- (  ) to serve patients better
- (  ) Integration between the hospital and primary care
- (  ) Learning, knowledge or professional development
- (  ) Clinical practice applicability
- (  ) Course content
- (  ) Course format
- (  ) Other reason. Please Specify: _____________________________

2. Com que frequência você acessou os conteúdos do Curso?

1. ( ) Apenas uma vez
2. ( ) 2-10vezes
3. (  ) 11 - 30 vezes
4. ( ) Acima de 30 vezes
5. ( ) Não acessei nenhuma vez → Porque você não acessou?

1. (  ) Não tentou
2. (   ) Não teve tempo
3. (   ) Não recebeu e-mail confirmando sua inscrição
4. (   ) Tentou acessar mas não conseguiu realizar o login
5. (  ) Estava sem acesso a internet

How often did you access the course contents?

1. ( ) Just once
2. ( ) 2-10 times
3. (  ) 11-30 times
4. ( ) More than 30 times
5. ( ) I did not access the course contents → Why not?
6. (  ) I did not try
7. (   ) I did not have time
8. (   ) I did not receive a confirmation email from my application
9. (   ) I tried to access but failed to login
10. (  ) I did not have access to the internet
11. Você teve dificuldade de conciliar as atividades do curso com o seu trabalho?
    - (  ) Sim. Por que?__________________________________
    - (  ) Não. Por que?__________________________________
    - Did you have difficulty balancing the course activities with your work?
    - (  ) Yes.Why?_______________________________________________
    - (  ) No. Why?________________________________________________
12. Você solicitou suporte técnico aos organizadores do curso?

( ) Não

( ) Sim → O seu problema foi resolvido? ( )Não

( ) Sim → Você acessou depois da solução? ( ) Sim

( ) Não. Por que?______________________________________

Did you ask for technical support from the course organizers?

( ) No

( ) Yes → The problem was solved? ( )No

( ) Yes → Did you access the course after the solution to your problem? ( ) Yes

( ) No. If not, why?______________

1. Os temas discutidos lhe despertaram atenção? ( ) Sim ( ) Não

Did the topics call your attention? ( ) Yes ( ) No

1. Qual o local que você tem acesso à internet?

a. ( ) Somente em casa

b. ( ) Somente no trabalho

c. ( ) Em casa e no trabalho

d. ( ) Outro. Qual?

________________________________________________________

In which location (s) you have internet access?

a. ( )Only at home

b. ( )Only at work

c. ( )At home and work

d. ( )Other. Please specify?_______________________________________

1. Você tem alguma sugestão de melhoria a ser realizada em uma próxima turma?____________________________________________

- Do you have any suggestions for any improvements for the next course?_________________________________________________________
- AS DEMAIS QUESTÕES SÃO APENAS PARA OS PROFISSIONAIS QUE ACESSARAM O CURSO

THE FOLLOWING QUESTIONS ARE ONLY ADDRESSED TO PROFESSIONALS WHO ACCESSED THE COURSE

1. Você acessou os Fóruns de Discussão?

- Módulo 1   ( )Sim ( ) Não. Se sim, participou? ( ) Sim ( ) Não

⮚Módulo 2  ( )Sim ( ) Não. Se sim, participou? ( ) Sim ( ) Não

⮚Módulo 3  ( )Sim ( ) Não. Se sim, participou? ( ) Sim ( ) Não

⮚Módulo 4  ( )Sim ( ) Não. Se sim, participou? ( ) Sim ( ) Não

⮚Módulo 5  ( )Sim ( ) Não. Se sim, participou? ( ) Sim ( ) Não

Did you enter into the dicussion forums?

- Module 1 ( )Yes ( ) No. If yes, did you participate? ( )Yes( )No
- Module 2 ( )Yes ( ) No. If yes, did you participate? ( )Yes( )No
- Module 3 ( )Yes ( ) No. If yes, did you participate? ( )Yes( )No
- Module 4 ( )Yes ( ) No. If yes, did you participate? ( )Yes( )No
- Module 5 ( )Yes ( ) No. If yes, did you participate? ( )Yes( )No

1. Qual a sua avaliação das Web conferências?

| Muito Ruim | Ruim | Nem ruim / Nem boa | Boa | Muito Boa | Não Participei |
| --- | --- | --- | --- | --- | --- |
| 1 | 2 | 3 | 4 | 5 | 0 |

What is your assessment of the web conferences?

| Very bad | Bad | Not bad / Not good | Good | Very Good | I did not take part |
| --- | --- | --- | --- | --- | --- |
| 1 | 2 | 3 | 4 | 5 | 0 |

1. Você assistiu as aulas?

- Módulo 1   ( )Sim ( ) Não. Se sim, quantas? 1 2 3 4 5 6 7 8

⮚Módulo 2  ( )Sim ( ) Não. Se sim, quantas? 1 2 3 4 5 6 7 8

⮚Módulo 3  ( )Sim ( ) Não. Se sim, quantas? 1 2 3 4 5 6 7 8

⮚Módulo 4  ( )Sim ( ) Não. Se sim, quantas? 1 2 3 4 5 6 7 8

⮚Módulo 5  ( )Sim ( ) Não. Se sim, quantas? 1 2 3 4 5 6 7

Did you attend the web conferences?

- Module 1 ( )Yes ( ) No. If yes, how many? 1 2 3 4 5 6 7 8
- Module 2 ( )Yes ( ) No. If yes, how many? 1 2 3 4 5 6 7 8
- Module 3 ( )Yes ( ) No. If yes, how many? 1 2 3 4 5 6 7 8
- Module 4 ( )Yes ( ) No. If yes, how many? 1 2 3 4 5 6 7 8
- Module 5 ( )Yes ( ) No. If yes, how many? 1 2 3 4 5 6 7

1. Qual a sua avaliação dos Casos Estruturados?

| Muito Ruim | Ruim | Nem ruim / Nem bons | Bons | Muito Bons | Não Participei |
| --- | --- | --- | --- | --- | --- |
| 1 | 2 | 3 |  4    | 5 | 0 |

What is your assessment of the structured clinical cases?

| Very bad | Bad | Not bad / Not good | Good | Very Good | I did not take part |
| --- | --- | --- | --- | --- | --- |
| 1 | 2 | 3 | 4 | 5 | 0 |

1. Qual sua avaliação das discussões que ocorreram nos Fóruns de Discussão?

| Muito Ruim | Ruim | Nem ruim / Nem boa | Boa | Muito Boa | Não Participei |
| --- | --- | --- | --- | --- | --- |
| 1 | 2 | 3 |  4    | 5 | 0 |

What is your assessment of the discussions that took place in our Discussion Forums?

| Very bad | Bad | Not bad / Not good | Good | Very Good | I did not take part |
| --- | --- | --- | --- | --- | --- |
| 1 | 2 | 3 | 4 | 5 | 0 |

1. Qual a sua avaliação da tutoria?

| Muito Ruim | Ruim | Nem ruim / Nem boa |   Boa |   Muito Boa | Não Participei |
| --- | --- | --- | --- | --- | --- |
| 1 | 2 | 3 | 4 | 5 | 0 |

What is your assessment of the course mentoring?

| Very bad | Bad | Not bad / Not good | Good | Very Good | I did not take part |
| --- | --- | --- | --- | --- | --- |
| 1 | 2 | 3 | 4 | 5 | 0 |
