# Supplementary material for: Implementation of integration strategies between primary care units and a regional general hospital in Brazil to update and connect health care professionals: a quasi-experimental study protocol
Source: BMC Health Serv Res. 2016 Aug 12;16:380. doi: 10.1186/s12913-016-1626-9 (PMC4983016; doi:10.1186/s12913-016-1626-9)
Supplement: Additional file 2: — Sociodemographic patient’s questionnaire. (DOCX 50 kb) [file 12913_2016_1626_MOESM2_ESM.docx]

Questionário sociodemográfico - Pacientes

Sociodemographic patient’s questionnaire

Número do paciente no estudo: P A __ __ __ __

*Patient’s Research Number: PA_ _ _ _*

Número do prontuário, no Hospital: ____________________________________

Patient’s hospital medical record number:________________________

Data do preenchimento do questionário: dd/mm/aaaa

Date that questionnaire was filled in: dd/mm/yyyy

Entrevista com o paciente

Patient interview

Lembretes:

Remarks:

-  Caro participante, na existência de desconforto ao responder alguma questão, a mesma poderá ser deixada em branco (sem resposta).

- - - - 1. - Dear participant, if you feel uncofortable in answering any question, you can leave it blank.

-  Os dados retirados deste questionário serão analisados, pela equipe de pesquisa do Hospital Municipal Moysés Deutsch – M’Boi Mirim, em conjunto com os dados de outros pacientes, garantindo o seu sigilo e privacidade.

- Data gathered from this questionnaire will be aggregated with that of other patients, by the research team of do Hospital Municipal Moysés Deutsch – M’Boi Mirim, so that individual data will not be visible, ensuring your confidentiality and privacy.

1. Qual a sua naturalidade (cidade onde nasceu):__________________________

Where are you originally from? (Birth City):_____________________________

Se não nasceu em São Paulo, há quantos anos mora em São Paulo?__________

If you were not born in São Paulo, how many years are you living here?

1. Qual é a sua etnia?
2. ( ) Branca
3. ( ) Preta
4. ( ) Amarela
5. ( ) Parda
6. ( ) Indígena

What is your ethnicity?

1. ( )White
2. ( )Black
3. ( )Yellow
4. ( )Brown
5. ( )Indigenous

4) Qual é o seu estado civil?

1. ( ) Solteiro (a)
2. ( ) Casado (a)
3. ( ) União consensual
4. ( ) Desquitado(a), divorciado(a) ou separado(a) judicialmente
5. ( ) Viúvo(a)

What is your marital status?

1. ( )Single
2. ( )Married
3. ( )Domestic partnership
4. ( )Divorced
5. ( )Widow or Widower

5) Quantas pessoas moram com você? (incluindo filhos, irmãos, parentes e amigos)?

1. ( )Moro sozinho
2. ( )Uma a três
3. ( )Quatro a sete
4. ( )Oito a dez
5. ( )Mais de dez

How many people live with you? (including your children, brothers and sisters, other relatives and friends)?

a.( ) I live alone

b.( ) One to three

c.( ) Four to seven

d.( ) Eight to ten

e.( ) More than ten

6) Como avalia a sua capacidade de enxergar? (se utiliza óculos ou lentes de contato, faça sua avaliação quando os estiver utilizando)

1. ( ) Incapaz
2. ( ) Grande dificuldade permanente
3. ( ) Alguma dificuldade permanente
4. ( ) Nenhuma dificuldade

How do you assess your vision? (if you use glasses or contact lenses, make your assessment with them on)

1. ( ) Unable
2. ( ) Big permanent difficulty
3. ( ) Some permanent difficulty
4. ( ) No difficulty

7) Como avalia a sua capacidade de ouvir? (se utiliza aparelho auditivo, faça sua avaliação quando o estiver utilizando)

1. ( ) Incapaz
2. ( ) Grande dificuldade permanente
3. ( ) Alguma dificuldade permanente
4. ( ) Nenhuma dificuldade

How do you assess your hearing? (if you use a hearing device, make your assessment while using it).

1. ( ) Unable
2. ( ) big permanent difficulty
3. ( ) Some permanent difficulty
4. ( ) No difficulty

8) Como avalia a sua capacidade de caminhar/subir escadas? (se utiliza prótese, bengala ou aparelho auxiliar, faça sua avaliação quando o estiver utilizando)

1. ( ) Incapaz
2. ( ) Grande dificuldade permanente
3. ( ) Alguma dificuldade permanente
4. ( ) Nenhuma dificuldade

How do you assess your ability to walk or climb stairs? (if you use a prosthesis, stick or auxiliary device, make your assessment when are using it).

1. ( ) Unable
2. ( ) big permanent difficulty
3. ( ) Some permanent difficulty
4. ( ) No difficulty

9) Você possui alguma das doenças abaixo?

Do you have any of the following diseases?

1. ( ) Hipertensão (Hypertension)
2. ( ) Diabetes  (Diabetes)
3. ( ) Artrite  (Arthritis)
4. ( ) Asma (Asthma)
5. ( ) Enfisema (Emphysema)
6. ( ) Depressão   (Depression)
7. ( ) AIDS (AIDS)
8. ( ) Osteoporose (Osteoporosis)
9. ( ) Insuficiência Cardíaca Congestiva  (Heart Failure)
10. ( ) Doença Coronariana (Coronary Heart Disease)
11. ( ) Dor (Chronic Pain)
12. ( ) Insuficiência Renal (Chronic Renal Failure)
13. ( ) Doença Vascular Periférica (Peripheral Vascular Disease)
14. ( ) Neuropatia Periférica  (Peripheral Neuropathy)
15. ( ) Demência (Dementia)
16. ( ) Ansiedade  (Anxiety)
17. ( ) Câncer. Se sim, qual? ______________________

Cancer. If yes, pelase, specify?_________________

10) Você é o provedor da sua família?

1. ( ) Sim
2. ( ) Não. Se não, quem é: _____________________(Grau de parentesco e idade)

Are you the principal income earner in the family?

1. ( )Yes
2. ( )No. If you are not, who is? _______________(Kinship degree and age).

11) Você está estudando atualmente?

1. ( ) Sim
2. ( ) Não

Are you currently studying?

1. ( )Yes
2. ( ) No

12) Qual é o curso mais elevado que você frequenta ou frequentou, no qual concluiu pelo menos uma série?

1. ( ) Alfabetização de adultos
2. ( ) Da 1ª à 4ª série do Ensino Fundamental (antigo primário)
3. ( ) Da 5ª à 8ª série do Ensino Fundamental (antigo ginásio)
4. ( ) Ensino médio (antigo 2o grau)
5. ( ) Ensino Superior (graduação)
6. ( ) Especialização, Mestrado ou Doutorado
7. ( ) Não estudei

Which is your highest education level achieved?

1. ( ) Adult literacy
2. ( ) From 1^st^ to 4^th^ grade (Elementary school)
3. ( ) From 5^th^ to 8^th^ grade (Middle school)
4. ( ) High School
5. ( ) Undergraduate Degree
6. ( ) Post Graduate Certificate, Master, Doctorate Degrees
7. ( ) I have not studied

Caso você não seja o provedor da família, responda a questão a seguir:

If you are not the principal income earner for you family, answer the following question:

13) Qual o nível de escolaridade do chefe da família?

1. ( ) Alfabetização de adultos
2. ( ) Da 1ª à 4ª série do Ensino Fundamental (antigo primário)
3. ( ) Da 5ª à 8ª série do Ensino Fundamental (antigo ginásio)
4. ( ) Ensino médio (antigo 2º grau)
5. ( ) Ensino Superior (graduação)
6. ( ) Especialização, Mestrado ou Doutorado
7. ( ) Não estudou
8. ( ) Não sei
9. ( ) Não se aplica

What is the highest level of education achieved by the principal income earner of your family?

1. ( ) Adult literacy
2. ( ) From 1^st^ to 4^th^ grade (Elementary school)
3. ( ) From 5^th^ to 8^th^ grade (Middle school)
4. ( ) High School
5. ( ) Undergraduate Degree
6. ( ) Post Graduate Certificate , Master or Doctorate Degree
7. ( ) He/She has not studied
8. ( ) I do not know
9. ( ) Not applicable

14) Você participou de alguma ação da sua comunidade no último ano?

1. ( )Sim
2. ( )Não

Have you volunteered in your community in the last year?

1. ( )Yes
2. ( )No

15) Você se sente seguro na sua vizinhança?

1. ( )Sim
2. ( )Somente de dia
3. ( )Somente à noite
4. ( )Não me sinto seguro

Do you feel safe in your neihghborhood?

1. ( )Yes
2. ( ) Only during the day
3. ( ) Only during the night
4. ( )No, I do not feel safe.

16) Você tem alguma rede de parentes ou amigos que lhe dão apoio em relação à sua doença?

1. ( )Sim
2. ( )Não

Do you have a support network, (relatives or friends) that help you with respect to your disease?

1. ( )Yes
2. ( )No

17) Você tem algum apoio de grupos não governamentais (ONGs, associações de moradores, etc.) em função da sua doença?

1. ( )Sim
2. ( )Não

Do you have support from non governmental groups (NGOs,neighborhood associations, etc.) related to your disease?

1. ( )Yes
2. ( )No

18) Quais são os meios de transporte que você mais utiliza?

1. ( ) À pé
2. ( ) Bicicleta
3. ( ) Ônibus
4. ( ) Carro
5. ( ) Motocicleta
6. ( ) Outros. Especificar: ___________________________________

What type of transportation do you typically use?

1. ( )Walking
2. ( )Bicycle
3. ( )Bus
4. ( )Car
5. ( )Motorcycle
6. ( )Other. Please,specify: ___________________________________

19) Quando você precisa fazer um exame ou ir à uma consulta na Unidade Básica de Saúde (UBS), qual o meio de transporte que você mais utiliza? (Apenas uma resposta)

1. ( ) À pé
2. ( ) Bicicleta
3. ( ) Ônibus
4. ( ) Carro
5. ( ) Motocicleta
6. ( ) Outros. Especificar: ___________________________________

When you need to go to the Primary Care Unit (PCU) for a consultation or to make an exam, what is your means of transportation? (Give only one answer).

1. ( ) Walking
2. ( ) Bicycle
3. ( ) Bus
4. ( ) Car
5. ( ) Motorcycle
6. ( ) Other. Please, specify: ___________________________________

20) Quando você precisa fazer um exame ou ir à uma consulta com especialista em outro serviço de saúde (Ambulatório de Especialidades, Centro de Reabilitação, CAPES, etc,), qual o meio de transporte que você mais utiliza? (Apenas uma resposta)

1. ( ) À pé
2. ( ) Bicicleta
3. ( ) Ônibus
4. ( ) Carro
5. ( ) Motocicleta
6. ( ) Outros. Especificar: ___________________________________

When you need to see a specialist for a consultation or make an exam at other health facilities (Specialized Ambulatory Care, Rehabilitation Center, Mental Health Unit, etc.), what is your means of transportation? (Just one answer).

1. ( )Walking
2. ( )Bicycle
3. ( )Bus
4. ( )Car
5. ( )Motorcycle
6. ( )Other. Specify: ___________________________________

21) Você já perdeu algum atendimento na UBS?

1. ( )Sim. Qual(is) motivo(s)?__________________________________
2. ( )Não

Have you ever missed any scheduled appointment at the PCU?

1. ( )Yes. Why(reasons)?__________________________________
2. ( )No

22) Você já perdeu algum atendimento em outro serviço de saúde (Ambulatório de Especialidades, Centro de Reabilitação, CAPES, etc,...)?

1. ( )Sim. Qual(is) motivo(s)?__________________________________
2. ( )Não

Have you ever missed any scheduled appointment at another health facility (Specialized Ambulatory Care, Rehabilitation Center, Mental Health Unit, etc.)?

1. ( )Yes. Why(reasons)?__________________________________
2. ( )No

23) Você possui convênio médico particular?

1. ( ) Sim. Qual?_______________
2. ( ) Não.

Do you have private health insurance?

1. ( )Yes. Which one?_______________
2. ( )No.

24) Qual a sua fonte de renda?

1. ( ) Aposentadoria e/ou pensão
2. ( ) Aluguel
3. ( ) Pensão alimentícia, mesada e/ou doação
4. ( ) Programas governamentais (programas oficiais de auxílio). Qual(is)?:_______
5. ( ) Salário/Trabalho
6. ( ) Seguro-desemprego
7. ( ) Outros. Qual: _______________________.
8. ( ) Não se aplica

What is your income source?

1. ( )Retirement and/or pension
2. ( ) Real state rent
3. ( ) Alimony, allowance and/or donation
4. ( ) Governmental Programs (official support programs).Which one(s)?:___
5. ( ) Salary/Work
6. ( ) Unemployment insurance
7. ( ) Other. Which one: ______________________________________________.

h.( )Not applicable

25) Você trabalha atualmente?

1. ( )Sim
2. ( )Não.

Are you currently working?

1. ( )Yes
2. ( )No.

26)  Se não, há quanto tempo está desempregado?___________________

If not, how long ago you are unemployed?___________________________

27)  Qual seu vínculo empregatício, atual ou no último trabalho?

1. ( ) CLT
2. ( ) Autônomo
3. ( ) Informal

Qual Atividade:________________________

Which is/was your employment status, in your current or last work?

1. ( ) CLT (Officially registered employment)
2. ( ) Self-employment
3. ( ) Informal employment

Which activity:________________________

28) Qual setor você trabalha ou trabalhou em seu último emprego?

1. ( ) Na agricultura, no campo, na fazenda ou na pesca.
2. ( ) Na indústria
3. ( ) Na construção civil
4. ( ) No comércio
5. ( ) No setor público
6. ( ) Prestação de Serviços
7. ( ) Serviço doméstico

In what economic sector you are currently working or did you work in your last job?

1. ( )In agriculture, countryside, farm or fishing.
2. ( )Industry
3. ( )Civil construction
4. ( )Commerce
5. ( )Public sector
6. ( )Service
7. ( )Domestic help

29) Quantas horas por semana você trabalha ou trabalhava em seu último emprego?

1. ( ) Sem jornada fixa, até 20 horas semanais
2. ( ) De 21 a 40 horas semanais
3. ( ) Mais de 40 horas semanais
4. ( ) Não se aplica

How long is your weekly work load work in your current or last job?

1. ( )Part-time, equal or less than 20 hours per week
2. ( )Between 21 and 40 hours per week
3. ( )More than 40 hours per week
4. ( )Not applicable

30) Com que idade você começou a trabalhar?

1. ( ) Antes dos 14 anos
2. ( ) Entre 14 e 16 anos
3. ( ) Entre 17 e 18 anos
4. ( ) Após 18 anos
5. ( )Não se aplica

What was your age when you began to work?

1. ( ) Less than 14 years old
2. ( ) Between 14 and 16 years old
3. ( ) Between 17 and 18 years old
4. ( ) More than 18 years old­­
5. ( ) Not applicable

31) Qual é o meio de transporte que você utiliza para ir ao trabalho?

1. ( ) À pé
2. ( ) Bicicleta
3. ( ) Ônibus
4. ( ) Carro
5. ( ) Motocicleta
6. ( ) Outros. Especificar:___________________________

What is your means of transportation to work?

1. ( )Walking
2. ( )Bicycle
3. ( )Bus
4. ( )Car
5. ( )Motorcycle
6. ( )Other. Please,specify: ___________________________________________

32)  Quanto tempo você demora somando a ida e a volta do trabalho?_________

How much time do you spend in transport to work round trip?______________

33)  Somando a renda das pessoas que moram com você, quanto é, aproximadamente, a renda familiar mensal?

What is the approximately income of your entire household?

1. ( ) No income
2. ( ) Equal to or less then one minimum salary (until R$ 724,00)
3. ( ) From 1 to 3 minimum salaries (from R$ 724,00 until R$ 2.172,00)
4. ( ) From 3 to 6 minimum salaries (from R$ 2.172,00 until R$ 4.344,00)
5. ( ) From 6 to 9 minimum salaries (from R$ 4.344,00 until R$ 6.516,00)
6. ( ) From 9 to 12 minimum salaries (from R$ 6.516,00 until R$ 8.688,00)
7. ( ) From 12 to 15 minimum salaries (de R$ 8.688,00 até R$ 10.860,00)
8. ( ) More than 15 minimum salaries (more than R$ 10.860,00)

***Minimum salary value in beginning of 2014.**

34) Qual é o tipo de domicílio que você mora:

1. ( )Apartamento
2. ( )Casa

What type of home do you live:

1. ( )Apartment
2. ( )House

35) De qual material o seu domicilio é feito:

1. ( ) Tijolo
2. ( ) Bloco
3. ( ) Madeira
4. ( ) Taipa
5. ( ) Outro: __________________________

What is the building material of your household

1. ( ) Fired brick
2. ( ) Concrete block
3. ( ) Wood
4. ( ) Mud and clay
5. ( ) Other: __________________________

36)  Quantos cômodos tem o domicílio que você mora (banheiro conta como 1 cômodo): _______________________________________________________

How many rooms there are in your household? (bathroom counts as a room, as well):_____________________________________________________

37)  O escoadouro do banheiro é ligado a:

1. ( )Sistema de esgoto (rede geral)
2. ( )Fossa séptica
3. ( )Fossa rudimentar
4. ( )Vala (céu aberto)
5. ( )Rio ou lago
6. ( )Outro. Qual:___________________________

What type of sewage system do you have:

1. ( )Sewage system
2. ( )Septic tank
3. ( )Rudimentary cesspit
4. ( )Open trench
5. ( )River or lake
6. ( )Other. Please, specify:___________________________

38) O lixo do seu domicílio é:

1. ( ) Coletado por serviço de limpeza. Qual a frequência semanal?:__________________
2. ( ) Colocado em caçamba de serviço de limpeza
3. ( ) Queimado (na propriedade)
4. ( ) Enterrado (na propriedade)
5. ( ) Jogado em terreno baldio ou logradouro
6. ( ) Jogado em rio ou lago
7. ( ) Tem outro destino. Qual:______________________________

The garbage of your house is:

1. ( ) Collected by public rubbish service. What is the weekly frequency?:________________________________________________
2. ( )Buckets (on the streets)
3. ( )Burned (in the house area)
4. ( )Buried (in the house area)
5. ( )Discarded on an empty lot or street
6. ( )Discarded in river or lake
7. ( )Other destination. Which one:______________________________

39) Você considera a sua alimentação:

1. ( )Pouco saudável
2. ( )Nem muito, nem pouco saudável
3. ( )Muito saudável

How do you consider your diet:

1. ( )Unhealthy
2. ( )More or less healthy
3. ( )Healthy

40) Você come alimentos fritos:

1. ( ) Todos os dias
2. ( ) Pelo menos uma vez na semana
3. ( ) Raramente
4. ( ) Nunca

You do eat fried foods:

1. ( )Everyday
2. ( )At least once a week
3. ( )Seldom
4. ( )Never

41) Você come frutas:

1. ( ) Todos os dias
2. ( ) Pelo menos uma vez na semana
3. ( ) Raramente
4. ( ) Nunca

You do eat fruits:

1. ( )Everyday
2. ( )At least once a week
3. ( )Seldom
4. ( )Never

42) Você come legumes e verduras:

1. ( )Todos os dias
2. ( ) Pelo menos uma vez na semana
3. ( )Raramente
4. ( )Nunca

You do eat vegetables

1. ( )Everyday
2. ( )At least once a week
3. ( )Seldom
4. ( )Never

43) Você pratica atividade física (qualquer movimento corporal acima do repouso pelo menos 30 minutos de forma contínua ou acumulada - 3x/10 min ou 2x/15 min):

1. ( ) Todos os dias
2. ( ) Pelo menos uma vez na semana
3. ( ) Raramente
4. ( ) Nunca

You do practice a physical activity (any body movement above rest for at least 30 minutes continuously or accumulated – 3x/10 min or 2x/15 min):

1. ( )Everyday
2. ( )At least once a week
3. ( )Seldom
4. ( )Never

44) Você pratica atividade física por que:

1. Faz parte do seu trabalho/ocupação.
2. Tem que andar muito para chegar ao local de trabalho.
3. Gosta muito de praticar exercícios físicos (jogar bola, andar, ir na academia).
4. Seu médico indicou.

Why do you practice physical activity?

1. Because of the nature of your work/occupation.
2. You have to walk far to get to the workplace
3. You like physical exercises(playing football, walking, to go to the gym).
4. Your doctor’s advice.

45) Você é tabagista (fumante)?

1. ( )Sim. Fumo há _____anos. Fumo aproximadamente ___ cigarros por dia.
2. ( )Não. Parei de fumar há ____ anos. Fumei durante____anos.
3. ( )Não. Nunca fumei.

Are you a smoker?

1. ( )Yes. I have smoked for _____years. I smoke ___ cigarettes per day.
2. ( )No. I stopped smoking for ____ years. I smoked for____years.
3. ( )No. I never smoked.

46) Qual a frequência do seu consumo de bebida alcoólica?

1. (  ) Nenhuma.
2. (  ) Uma ou menos de uma vez por mês.
3. ( ) 2 a 4 vezes por mês.
4. (  )2 a 4 vezes por semana.

e. ( ) 4 ou mais vezes por semana.

What is your frequency of alcohol intake?

1. (  ) None.
2. (  ) Once or less per month
3. ( ) 2 to 4 times per month.
4. (  )2 to 4 times per week.

e.( ) More than 4 times per week.

47) Você faz uso de outras drogas?

1. ( )Sim. Qual(is)_____________________________________
2. ( )Não, pois parei há ____ anos.
3. ( )Não. Nunca usei.

Do you use other drugs?

1. ( )Yes. Which one (s)?_____________________________________
2. ( )No, I stopped drug using for____ years.
3. ( )No. I never used drugs.
