# Supplementary material for: Implementation of integration strategies between primary care units and a regional general hospital in Brazil to update and connect health care professionals: a quasi-experimental study protocol
Source: BMC Health Serv Res. 2016 Aug 12;16:380. doi: 10.1186/s12913-016-1626-9 (PMC4983016; doi:10.1186/s12913-016-1626-9)
Supplement: Additional file 3: — Questionnaire on treatment adherence. (DOCX 72 kb) [file 12913_2016_1626_MOESM3_ESM.docx]

Questionário de adesão ao tratamento

*Questionnaire on treatment adherence*

Número do paciente na pesquisa : P A __ __ __ __

*Patient’s Research Number*

Data da visita na UBS ou Domicílio: dd/ mm /yyyy

*Interview date at Patient’s home or at the Primary Care Unit*

1. Quais medicamentos você está tomando atualmente (nome, dose e aprazamento)?

*Which medications are you currently using (name, doses, and frequency)?*

A.________________________________ B.________________________________ C.________________________________ D.________________________________ E.________________________________ F.________________________________

1. Desses medicamentos, quais o paciente e os familiares dizem que ele toma corretamente de acordo com a tabela abaixo:

*From the point of view of the patient or caregiver,*

*which of the following medications are being correctly administered:*

|  | A   | B | C | D | E | F |
| --- | --- | --- | --- | --- | --- | --- |
| Você toma o medicamento todos os dias?  *Have you take this medicine everyday?* | ( )S ( )N  *( )Y ( )N* | ( )S ( )N  *( )Y ( )N* | ( )S ( )N  *( )Y ( )N* | ( )S ( )N  *( )Y ( )N* | ( )S ( )N  *( )Y ( )N* | ( )S ( )N  *( )Y ( )N* |
| Você consegue tomar o medicamento nos horários corretos?  *Are you taking this medicine at the correct times?* | ( )S ( )N  *( )Y ( )N* | ( )S ( )N  *( )Y ( )N* | ( )S ( )N  *( )Y ( )N* | ( )S ( )N  *( )Y ( )N* | ( )S ( )N  *( )Y ( )N* | ( )S ( )N  *( )Y ( )N* |
| O medicamento está disponível em casa?  *Is the medicine is available in home?* | ( )S ( )N  *( )Y ( )N* | ( )S ( )N  *( )Y ( )N* | ( )S ( )N  *( )Y ( )N* |   ( )S ( )N  *( )Y ( )N*   | ( )S ( )N  *( )Y ( )N* | ( )S ( )N  *( )Y ( )N* |
| A cartela/frasco do medicamento indica que este está sendo consumido?  *The medicine packaging indicates that it is being consumed?* | ( )S ( )N  *( )Y ( )N* | ( )S ( )N  *( )Y ( )N* | ( )S ( )N  *( )Y ( )N* | ( )S ( )N  *( )Y ( )N* | ( )S ( )N  *( )Y ( )N* | ( )S ( )N  *( )Y ( )N* |
| Sente algum desconforto? Se sim, qual?  *Do you feel any discomfort taking this medicine?*  *If yes, which one?* | ( )S ( )N  *( )Y ( )N* | ( )S ( )N  *( )Y ( )N* | ( )S ( )N  *( )Y ( )N* | ( )S ( )N  *( )Y ( )N* | ( )S ( )N  *( )Y ( )N* | ( )S ( )N  *( )Y ( )N* |
| Percebe algum efeito colateral? Se sim, qual?  *Do you notice any side effect taking this medicine?*  *If yes, which one?* | ( )S ( )N  *( )Y ( )N* | ( )S ( )N  *( )Y ( )N* | ( )S ( )N  *( )Y ( )N* | ( )S ( )N  *( )Y ( )N* | ( )S ( )N  *( )Y ( )N* | ( )S ( )N  *( )Y ( )N* |

1. Quais as dificuldades em tomar corretamente os medicamentos prescritos? (pode preencher mais de uma opção).

*What difficulties are there in properly taking the prescribed medicines? (You can fill more than one option).*

(  ) Esquece que precisa tomar.

- *Forget that you need to take it.*
- (  ) Esquece que já tomou.
- *Forgot that you took it.*
- (  ) São muitos medicamentos para lembrar (Polifarmácia).

Too many medicines to remember (Polipharmacy).

- (  ) Desconfortos.
- Discomforts.
- (  ) Efeitos colaterais.
- Side effects.
- 4. Algum profissional de saúde (HMMD ou UBS) lhe fez alguma recomendação sobre mudança de hábitos? Qual(is)?_______________________________________________________________________________________________

*Have any health professionals from the hospital or the Primary Care Unit recommended you to change habits? Which ones?________________________________________*

| Recomendação  Recommendation | Alimentar  Eating | Atividade Física  Physical activity | Tabagismo  Smoking | Álcool  Alcohol | Drogas  Drugs | Outra recomendação Qual?  Any other rec? If yes, which one? |
| --- | --- | --- | --- | --- | --- | --- |
| Foi feita a recomendação?  Have you received a recommendation? | ( )S ( )N  *( )Y ( )N* | ( )S ( )N  *( )Y ( )N* | ( )S ( )N  *( )Y ( )N* | ( )S( )N  *( )Y( )N* | ( )S ( )N  *( )Y( )N* | ( )S ( )N  *( )Y ( )N* |
| Paciente, o quanto você mudou os seus hábitos?  Have you changed this habit? | ( )Nada  ( )Pouco  () Muito | ( )Nada  ( )Pouco  ( )Muito | ( )Nada  ( )Pouco  ( )Muito | ( )Nada  ( )Pouco  ( )Muito | ( )Nada  ( )Pouco  ( )Muito | ( )Nada  ( )Pouco  ( )Muito |
|  | ( )Nothing  ( ) Some  ( ) A lot | ( )Nothing  ( ) Some  ( ) A lot | ( )Nothing  ( ) Some  ( ) A lot | ()Nothing  ( ) Some  ( ) A lot | ( )Nothing  ( ) Some  ( ) A lot | ( )Nothing  ( ) Some  ( ) A lot |

O que mudou?

What has changed?

A(E).________________________________________________________AF(PA).______________________________________________________T(S).________________________________________________________ALC(ALC).____________________________________________________

D(D).________________________________________________________

5. Nos últimos 12 meses, precisou ir a algum serviço de pronto-atendimento (Pronto-socorro, AMA, UBS demanda espontânea)?

( ) Não ( ) Sim. Por quê?_____________________________________

In the last 12 months, have you needed to see a doctor in any emergency facility (Emergency room or Primary Care Unit)?

( )No ( ) Yes. Why?________________________________________________

6. Nos últimos 12 meses você foi internado alguma outra vez no HMMD ou em outro hospital?

( ) Sim ( ) Não.

Se sim, pelo mesmo motivo ou por outro?___________________________

Qual motivo?__________________________________________________

Quantas vezes?________________________________________________

In the last 12 months, have you been readmitted at the HMMD or another hospital?

If yes, were you readmitted for the same or different reasons as the reference hospitalization?_____________________________________

Which reason?______________________________________________________

How many readmissions?_____________________________________________

7. Você acha que seu estado geral de saúde melhorou, piorou, ou está igual ao da época da ultima alta hospitalar no HMMD?

( )Muito Melhor ( )Melhor ( ) Nem melhor nem Pior ( ) Pior ( ) Muito Pior

Do you think that your overall health has been improved, impaired or as same as it was related to the reference hospitalization at HMMD.

( )Much Better ( ) Better ( ) As same as ( ) Worse ( ) Much worse
