# Supplementary material for: Implementation of integration strategies between primary care units and a regional general hospital in Brazil to update and connect health care professionals: a quasi-experimental study protocol
Source: BMC Health Serv Res. 2016 Aug 12;16:380. doi: 10.1186/s12913-016-1626-9 (PMC4983016; doi:10.1186/s12913-016-1626-9)
Supplement: Additional file 4: — Socio-demographic health professional questionnaire. (DOCX 38 kb) [file 12913_2016_1626_MOESM4_ESM.docx]

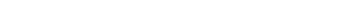


Questionário sociodemográfico dos profissionais de saúde.

Socio-demographic health professional questionnaire.

Número do sujeito de pesquisa: P R __ __ __ __ (números)

Professional’s research number: PR_ _ _ _ (numbers)

Data de preenchimento do questionário: dd/mm/aaaa

Date questionnaire was filled in: dd/mm/yyyy

Lembretes:

Remarks:

- -  Caro participante, na existência de desconforto ao responder alguma questão, a mesma poderá ser deixada em branco (sem resposta).
  - - - 1. - Dear participant, if you feel uncomfortable in answering any question, you can leave it blank.
- -  Os dados retirados deste questionário serão analisados, pela equipe de pesquisa do Hospital Municipal Moysés Deutsch – M’Boi Mirim (HMMD), em conjunto com os dados de outros profissionais, garantindo o seu sigilo e privacidade.
- - The data gathered from this questionnaire will be analysed by the rersearch team of the Hospital Municipal Moysés Deutsch – M’Boi Mirim (HMMD), together with data from other professionals, ensuring their confidentiality and privacy.
- Perguntas:
- Questions:

1. Idade: _______anos

Age:_______years

1. Sexo: ( ) Masculino ( ) Feminino

Gender: ( ) Male ( ) Female

1. Situação familiar: ( ) Com parceiro estável ( ) Sem parceiro estável

Domestic Status: ( )with domestic partner( )without domestic partner

1. Número de filhos sob sua responsabilidade:__________________

Number of Children under your responsibility:______________

1. É profissional do Hospital ou UBS?

a. ( ) HMMD. Qual setor?_________________________________

b. ( ) UBS. Qual?_______________________________________

Are you currently working at the HMMD or PCU?

a.( )HMMD. Which sector?________________________________________

b.( )PCU. Which one?_____________________________________

6)  Quando começou a atuar nessa instituição? mm/aaaa

When did you begin to work at this institution? mm/yyyy

7)  Qual o cargo atual? ______________________________

What is your current position?__________________________

8)  Quantas horas semanais você trabalha nessa instituição? (incluindo reuniões, atividades formativas, plantões) _______horas

How many hours per week do you work at this institution? (Including meetings, educational activities, duties, and so on)____hours

9) Período de trabalho:

Daily work load:

( ) Full day ( ) Half day

10) Contrato de trabalho:

( ) Temporário ( ) Fixo: CLT ( ) / Estatutário ( )

Labor contract:

( )Temporary ( )Fixed: Officially registered (CLT) ( ) / Public employee( )

11) Número total de anos trabalhados até hoje (somando todos os empregos que teve até hoje):_____________________________________

Total number of years worked until present (entire employment history)___________________________________________________________

12) Histórico Escolar:

Educational History:

| Curso  Educational Type | Realizou?  HIstory | Qual?  Specify | Ano de Conclusão  Year completed |
| --- | --- | --- | --- |
| Curso Técnico  Technical Education |   ( ) Sim ( ) Não  ( )Yes ( )No |  |  |
| Undergraduate Education | ( ) Sim ( ) Não   ( )Yes ( )No |  |  |
| Residência  Internship | ( ) Sim ( ) Não  ( )Yes ( )No |  |  |
| Especialização  Certificate (not degree earning) | ( ) Sim ( ) Não  ( )Yes ( )No |  |  |
| Pós-Graduação  Post-Graduation |  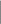  ( ) Sim ( ) Não  ( )Yes ( )No |  | 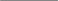 |

13) Número total de anos trabalhados até hoje (somando todos os empregos que teve até hoje):_____________________________________

Total number of years worked until present (entire employment history)___________________________________________________________

14) Já realizou outros cursos de Aprimoramento? ( )Sim ( )Não

Have you completed other professional development courses?

( )Yes ( )No

| Se sim, qual(s)?  If yes, which one(s)? | Considera que o(s) curso(s) tenha(m) acrescentado conteúdo relevante para sua experiência profissional?  Do you consider that this course(s) has contributed to your professional development? | Conseguiu aplicar na prática o conteúdo adquirido no curso?  Were you able to apply your course learning to your job? |   Ano de conclusão  Year completed |
| --- | --- | --- | --- |
| Course | ( )Sim ( )Não  ( )Yes ( )No | ( )Sim ( ) Não   ( )Yes ( )No |  |
| Course | ( )Sim ( )Não  ( )Yes ( )No | ( )Sim ( ) Não  ( )Yes ( )No |  |
| Course | ( )Sim ( )Não  ( )Yes ( )No | ( )Sim ( ) Não  ( )Yes ( )No   |  |
| Course | ( )Sim ( )Não  ( )Yes ( )No | ( )Sim ( ) Não  ( )Yes ( )No |  |

15)  Qual(s) fonte(s) de informação você utiliza para a tomada de decisões na sua prática clínica?

( ) Livros

( ) Revistas Científicas

( ) Protocolos clínicos institucionais

( ) Consulta a colegas de profissão

( ) Redes Sociais

( ) Internet

( ) Outra(s).Qual(s)?_______________________________________

What information source(s) do you use to support your clinical decisions?

( ) Books

( ) Scientific journals

( ) Institutional guidelines or protocols

( ) Colleague Consultation

( ) Social networks

( ) Internet

( ) Other.Which one(s)?_______________________________________

16)  Qual o seu grau de satisfação com as informações que você tem acesso para a tomada de decisão clínica?

What is your level of satisfaction with the information that supports your clinical decisions?

| Muito insatisfeito  Very unsatisfied | Insatisfeito  unsatisfied | Nem satisfeito nem insatisfeito  Neither satisfied nor unsatisfied | Satisfeito  Satisfied | Muito satisfeito  Very Satisfied |
| --- | --- | --- | --- | --- |
| 1 | 2 | 3 | 4 | 5 |

17)  Qual importância você dá à experiência prática profissional para a tomada de decisão clínica?

What level of importance do you give to practical experience to support your clinical decisions?

| Nenhuma  None | Pouca  Little | Nem pouca nem muita  Neutral | Muita  Much | Extrema  Very Much |
| --- | --- | --- | --- | --- |

18)  Qual importância você dá às recomendações baseadas em evidências para a tomada de decisão clínica?

What level of importance do you give to evidence-based recommendations to support your clinical decisions?

| Nenhuma  None | Pouca  Little | Nem pouca nem muita  More or less | Muita  Much | Extrema  Very Much |
| --- | --- | --- | --- | --- |
| 1 | 2 | 3 | 4 | 5 |

19)  Qual importância que você dá à existência de protocolos institucionais para tomada de decisão clínica?

What level of importance do you give to institutional guidelines or protocols support your clinical decisions?

| Nenhuma  None | Pouca  Little | Nem pouca nem muita  More or less | Muita  Much | Extrema  Very much |
| --- | --- | --- | --- | --- |
| 1 | 2 | 3 | 4 | 5 |

20)  Qual importância você dá às características do paciente na adesão a tratamentos para a tomada de decisão clínica?

What level of importance do you give to patient treatment adherence to support your clinical decisions?

| Nenhuma  None | Pouca  Little | Nem pouca nem muita  More or less | Muita  Much | Extrema  Very much |
| --- | --- | --- | --- | --- |
| 1 | 2 | 3 | 4 | 5 |

21)  Qual o seu nível de dificuldade para obter informações científicas que possam auxiliar em tomadas de decisões clínicas?

How difficult is it to obtain scientific information to support clinical decisions?

| Nenhuma  None | Pouca dificuldade  Little difficulty | nem pouca nem muita  More or less | Alguma dificuldade  Some difficulty | Muita dificuldade  Very difficult |
| --- | --- | --- | --- | --- |
| 1 |   2 | 3 | 4 | 5 |

22)  Qual seu nível de satisfação com as informações que são compartilhadas entre o HMMD e as UBS, na tomada de decisão clínica?

How satisfied are you with the information shared by the HMMD and the PCUs, to support your clinical decisions?

| Muito insatisfeito  Very unsatisfied | Insatisfeito  unsatisfied | Nem satisfeito nem insatisfeito  Nor satisfied neither unsatisfied | Satisfeito  Satisfied | Muito satisfeito  Very Satisfied |
| --- | --- | --- | --- | --- |
| 1 | 2 | 3 |   4   |   5   |

23)  Qual seu nível de satisfação com as informações que são compartilhadas entre a sua equipe, tanto no HMMD quanto na UBS, na tomada de decisão clínica?

What is your satisfaction level with the information shared by your own team, in HMMD and PCU, in making your clinical decision?

| Muito insatisfeito  Very unsatisfied | Insatisfeito  unsatisfied | Nem satisfeito nem insatisfeito  Nor satisfied neither unsatisfied | Satisfeito  Satisfied | Muito satisfeito  Very Satisfied |
| --- | --- | --- | --- | --- |
| 1 | 2 | 3 |   4   |   5   |

24)  Qual importância você dá às opiniões de outros membros da sua equipe, na tomada de decisão clínica?

What level of importance do you give to the opinions from your team members, in making your clinical decisions?

| Nenhuma  None | Pouca  Little | Nem pouca nem muita  More or less | Muita  Much | Extrema  Very Much |
| --- | --- | --- | --- | --- |
| 1 | 2 | 3 | 4 | 5 |

25) Qual importância você dá às opiniões dos pacientes, na tomada de decisão clínica?

What level of importance do you give to your patient’s opinion, in your clinical decisions?

| Nenhuma  None | Pouca  Little | Nem pouca nem muita  More or less | Muita  Much | Extrema  Very much |
| --- | --- | --- | --- | --- |
| 1 | 2 | 3 | 4 | 5 |

26)  Qual seu nível de satisfação com a assistência que você tem prestado aos seus pacientes?

What is your satisfaction with the overall service level you are providing to your patients?

| Muito insatisfeito  Very unsatisfied | Insatisfeito  unsatisfied | Nem satisfeito nem insatisfeito  Nor satisfied neither unsatisfied | Satisfeito  Satisfied | Muito satisfeito  Very Satisfied |
| --- | --- | --- | --- | --- |
| 1 | 2 | 3 |   4   |   5   |

27)  Qual sua expectativa em relação ao Curso EAD que estamos iniciando?

What is your level of expectation for the course that we are undertaking?

| Muito baixa  Very low | Baixa  Low | Nem baixa nem alta  Neutral | Alta  High | Muito alta  Very high |
| --- | --- | --- | --- | --- |
| 1 | 2 | 3 |   4   |   5   |

28) Nível médio de salário mensal: R$______________

Average monthly salary: R$ (Brazilian currency)_________

29) Tem dificuldades econômicas ao chegar no final do mês?

Do you have economic difficulties?

| Nunca  Never | Algumas vezes  Sometimes | Muitas vezes  Many times | Quase sempre  Almost always | Sempre  Always |
| --- | --- | --- | --- | --- |
| 1 | 2 | 3 | 4 | 5 |

30) Durante o último ano, você teve que se ausentar do trabalho por motivo de doença?

During last year, did you have to be absent from work because of illness?

( ) Sim. Quantos dias, aproximadamente: _________ dias.

( ) Não

( ) Yes. How many days, approximately: _________ days.

( ) No
